# Link Definition Ameliorating Community Detection in Collaboration Networks

**DOI:** 10.3389/fdata.2019.00022

**Published:** 2019-06-26

**Authors:** Saharnaz Dilmaghani, Matthias R. Brust, Apivadee Piyatumrong, Grégoire Danoy, Pascal Bouvry

**Affiliations:** ^1^Interdisciplinary Centre for Security, Reliability, and Trust (SnT), University of Luxembourg, Esch-sur-Alzette, Luxembourg; ^2^National Electronics and Computer Technology Center, A Member of NSTDA, Bangkok, Thailand

**Keywords:** network interactions, data-to-network, collaboration network, data analysis, community detection analysis

## Abstract

Collaboration networks are defined as a set of individuals who come together and collaborate on particular tasks such as publishing a paper. The analysis of such networks permits to extract knowledge on the structure and patterns of communities. The link definition and network extraction have a high impact on the analysis of collaboration networks. Previous studies model the connectivity in a network considering it as a binomial problem with respect to the existence of a collaboration between individuals. However, such a data consists of a high diversity of features that describe the quality of the interaction such as the contribution amount of each individual. In this paper, we have determined a solution to extract collaboration networks using corresponding features in a dataset. We define *collaboration score* to quantify the collaboration between collaborators. In order to validate our proposed method, we benefit from a scientific research institute dataset in which researchers are co–authors who are involved in the production of papers, prototypes, and intellectual properties (IP). We evaluated the generated networks, produced through different thresholds of *collaboration score*, by employing a set of network analysis metrics such as clustering coefficient, network density, and centrality measures. We investigated more the obtained networks using a community detection algorithm to further discuss the impact of our model on community detection. The outcome shows that the quality of resulted communities on the extracted collaboration networks can differ significantly based on the choice of the linkage threshold.

## 1. Introduction

Collaboration networks are social structures which indicate the relationship between collaborators who perform on the same tasks. Collaboration is an essential component to define the success of today's knowledge sharing ecosystem (Huang et al., 2008) and establishment of innovation. In collaboration networks, nodes represent individuals (aka collaborators) and links between them imply a collaboration. The analysis of collaboration networks can reveal information about the most likely behavior of individuals and groups in the network (Jamali and Abolhassani, 2006) such as discovering the interaction patterns (Akbas et al., 2013; Long et al., 2014; Dilmaghani et al., 2019), the evolution of collaboration communities (Kibanov et al., 2013) and predictive models on the productivity and longevity of collaborations (Chakraborty et al., 2015).

One prominent property studied in the context of collaboration networks is the community structure of nodes (Pan et al., 2014). The discovery of communities, with dense intra-connections and comparatively sparse inter-cluster, can be beneficial for various applications such as discovering common research area of potential collaborators (Bedi and Sharma, 2016). Various network-based community detection algorithms are used for this purpose, e.g., *Louvain*'s algorithm (Blondel et al., 2008), Label Propagation Algorithm (LPA) (Zhu and Ghahramani, 2002).

Most collaboration data are stored in relational databases which are used to extract the collaboration networks to perform network analysis. The context of scientific collaboration networks has been initiated with the studies of Newman (2001a) and Newman (2001b). The network is defined such that the researchers are represented as nodes and the links constructed if at least one paper happened to be published by them. Other studies such as Chakraborty et al. (2015) have followed a similar generative approach to construct the collaboration network from the dataset. In a recent study (Sharma and Bhavani, 2019), a weighted scientific collaboration network has been proposed such that links are weighted by the number of papers. One drawback of previous studies is the elimination of other potential features that represent the collaborations (e.g., date, number of citations). The information which is attached to the data can substantially impact the underlying network representation and, therefore, the outcomes of network analysis (e.g., community detection). Thus the appropriate use of network analysis, substantially depends on choosing the right network representation (Scholtes, 2017), i.e., the definition of nodes and links (Butts, 2009). Besides, in some cases, the definition of the link also requires determining a *threshold* which can significantly alter the outcomes of network properties, e.g., network density (Faust, 2007).

In this paper, we investigated the definition of the fundamental research question of how and which network representation to choose for a given set of data. The drawback of previous studies is that they only consider the existence of a collaboration between individuals to connect them in the network. However, our work proposes a standardized method to produce networks from large and complex datasets. We define a method to construct scientific collaboration networks from the data considering different features describing the collaboration. Furthermore, we benefit from the scientific collaboration dataset of *National Electronics and Computer Technology Center* (NECTEC) to examine our method. Interestingly, our results indicate that identifying a network construction model leads to a less noisy yet well–shaped community structure network with high modularity score.

## 2. Dataset

We benefit from a particular collaboration database provided by the *National Electronics and Computer Technology Center* (NECTEC) that presents different projects and collaborations in the area of R&D^1^. The whole database is the knowledge management about projects within distinct deliverables where the key information is to know project contributors and contributions. The database consists of three datasets, each indicates a particular deliverable: *PAPER, PROTOTYPE*, and *IP* (intellectual property) conducted between July 2013 and July 2018.

The datasets of combined research teams information consist of approximately 8,000 records which correspond to the information of more than 2,300 projects. Detailed statistical information regarding each dataset is provided in Table 1. Overall, NECTEC has more than 1,000 members who are contributing to different deliverables with certain features that have been evaluated by the organization. For each researcher who collaborated on a contribution, a contribution percentage has been recorded. Another feature named IC–score which is designed by NECTEC, evaluates the scientific value and the outcome of contributions. For instance, producing a prototype in an industrial stage has a higher impact than one in the laboratory stage. For each project, the IC–score is divided between each contributor considering their individual participation in the project. Overall, each dataset of the deliverables contains (a) project ID, (b) collaborator's ID, (c) contribution percentage of a collaborator for each project, (d) IC–score of a collaborator for each project.

**Table 1:** General overview of the datasets from NECTEC.

**Deliverable type**	**# Researchers**	**# Projects**	**Cont. percentage**	**IC–score**
*PAPER*	576	1717	*μ* = 22.22, σ = 19.73	*μ* = 3.89, σ = 4.61
*PROTOTYPE*	524	539	*μ* = 15.54, σ = 13.73	*μ* = 9.41, σ = 10.75
*IP*	489	630	*μ* = 25.15, σ = 24.42	*μ* = 4.08, σ = 4.63
Total	1, 056	2, 347	*μ* = 20.78, σ = 19.82	*μ* = 5.81, σ = 7.73

*Contribution percentage (Cont. percentage) and IC–score are features extracted from the dataset and describe the collaboration*.

## 3. Methodology for Link Construction

We propose a *collaboration score* function that takes into account the combination of features extracted from the dataset. The purpose is to quantify the contribution of researchers considering features describing the collaborations. The collaboration score is the key element to define the link in the network while nodes are co–authors. We introduce a *linkage threshold* (*LT*) on obtained collaboration scores. Thus, multiple networks are produced using various *LT* values.

We define the *collaboration score* function based on the features extracted from the NECTEC datasets which includes (a) the number of projects, (b) the contribution percentage of researchers, and (c) the IC–score of researchers. Given two researchers *i* and *j* worked on a mutual project *p*, i.e., (*i, j*), let *n* be the number of projects that *i* and *j* have collaborated, and *p*_*k, i*_ and *p*_*k, j*_ represent the contribution percentage of researcher *i* and *j*, respectively, for the *k*th project. Likewise, *s*_*k, i*_ and *s*_*k, j*_ indicate the IC–score of each researcher on the *kth* project. Hence, we determine the *collaboration score* function as follows.

(1) $$\begin{array}{r} f_{i,j}=\frac{1}{n}\left(\frac{1}{2}\sum_{k=1}^{n}\left(p_{k,i}+p_{k,j}\right)+\frac{1}{2}\sum_{k=1}^{n}\left(s_{k,i}+s_{k,j}\right)\right) \end{array}$$

The function takes into account the average of IC–score and contribution percentage between any tuple of collaborators. The *LT*, then, is defined such that it determines different levels of collaboration score in the network. The range of *LT* varies from 0 to 1, which is the normalized range of collaboration score. In a nutshell, increasing *LT* enlarges the number of collaborations.

The threshold values indicate links in the network between the nodes. We produce a set of networks considering various *LT*s. Algorithm 1 shows the pseudocode of the data transformation to networks. A relational dataset of collaborations is the input of the algorithm. The researchers are determined as nodes of the network. For each tuple of researchers, the collaboration score is measured (see line 4). In order to generate a network, links are produced considering a particular *LT* value. All collaborations that are less or equal than the level of the chosen threshold are determined as links in the network (see line 7). Considering various levels of *LT*, a set of networks is generated by the algorithm which is examined in section 4.

**Algorithm 1:** Network Extraction from Data

**Input:** *D*, scientific collaboration dataset **Output:** G, a vector of generated networks
1: **procedure** TRANSFORM-TO-NETWORK(*D*)
2: *colList* ← researchers from *D*
3: **for** *tuple*(*i, j*) in *colList* **do**
4: *f*.append←*collaborationScore*(*tuple*(*i, j*))
5: *collaboration*.append← Concatenate *tuple*(*i, j*) and *normalize*(*f*)
6: **for** *LT* in **range**(*normalize*(*f*)) **do**
7: **if** *collaboration*.*normalize*(*f*) ≤ *LT* **then**
8: *nodes*.append([*i, j*])
9: *links*.append([*tuple*(*i, j*)])
10: G ← *Network*(*nodes, links*)
11: G.append *G*
12: return G

## 4. Results

Our proposed method has been employed on different deliverable types of the previously described NECTEC collaboration data. As a result of the extraction process, our method returns a set of corresponding collaboration networks. In the first stage, we exploit the distribution of the collaboration score (*f*) within each dataset. Next, we analyze the topology of the extracted networks given the different values of *LT* by measuring a set of network metrics. Furthermore, for each generated network, we identify the communities using the *Louvain* algorithm and evaluate their quality.

### 4.1. Data Processing

**Figure 1:**
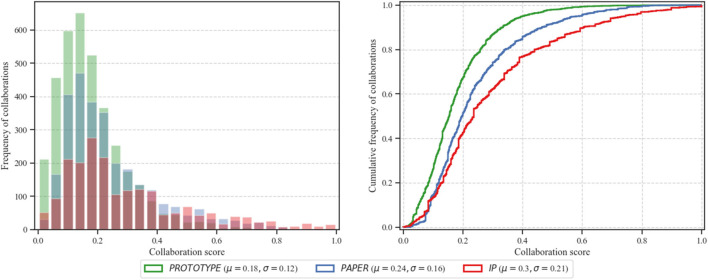
The histogram and cumulative distribution function (CDF) of generated collaboration score (*f*).

We exploit the histogram and cumulative distribution function (CDF) of *f* for each dataset of deliverables from NECTEC. Figure 1 describes the frequency and distribution of the obtained *f* after normalization. The average (μ) of *f* for *PAPER, PROTOTYPE*, and *IP* are 0.24 [standard deviation (σ = 0.16)], 0.18 (σ = 0.12), and 0.3 (σ = 0.21), respectively. Furthermore, the figure also shows that the majority of collaborators have relatively low number of contribution. Nevertheless a small number of collaborators are strongly collaborating in various projects.

### 4.2. Topological Analysis

**Figure 2:**
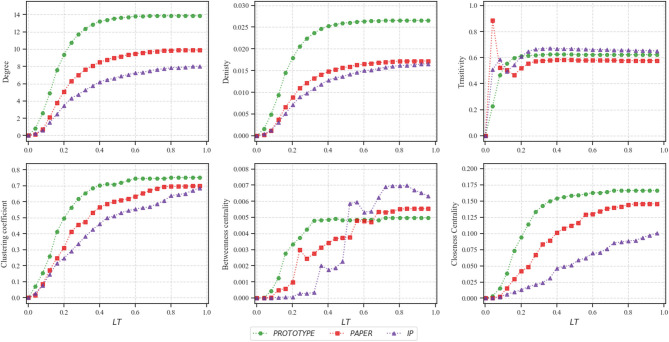
Topological analysis of a set of 41 produced networks from each dataset while increasing *LT* from 0 to 1 by 0.025.

We analyze the topology and structure of extracted networks from each dataset by calculating a set of network metrics: degree, network density, transitivity, clustering coefficient, betweenness centrality, and closeness centrality. Figure 2 describes the evolution of these metrics on a set of 41 networks while increasing *LT* from 0 to 1 with the step of 0.025.

The degree of a node in collaboration networks represents the number of direct collaborations for each individual. The average node degree of networks obtained from *PAPER* is 6.59, *PROTOTYPE* is 11.46, and *IP* is 5.71 which indicates that on average, teams in *PROTOTYPE* had significantly higher collaborations compared to others. As illustrated in Figure 2, the degree of extracted networks does not change significantly. The reason is after a certain threshold of *LT*, the number of new links which have been added to the network does not grow significantly while the number of nodes stays constant. A similar scenario occurs when measuring network density. The network density calculates the ratio of existing links to the number of all possible links in a network such that a density close to 0 identifies a sparse network while a density equal to 1 is a complete network. With *LT* close to zero, the network mostly consists of isolated nodes which explains why in all three datasets the network density is close to zero. Eventually, the density of the network increases slowly and remains steady. The reason is due to the high number of nodes compared to the number of collaborations between the nodes. This indicates the fact that in real-world collaboration networks each collaborator may only collaborate with a small number of collaborators, hence, the networks are considered as rather sparse.

In order to get knowledge on the complexity of collaborations of each dataset, we calculate the transitivity and clustering coefficient of networks. Transitivity refers to the extent to which the relation that relates two nodes in a network that are connected by a link is transitive. Thus, it represents the symmetry of collaborations in our networks and forms triangles of collaborations. Figure 2 illustrates fluctuations for networks constructed with lower *LT*, however, quickly it approaches a consistent value.

On the other hand, the clustering coefficient describes the likelihood of nodes in a network that tend to cluster together (Watts and Strogatz, 1998). The average clustering coefficient of produced networks is 0.44 for *PAPER*, 0.61 for *PROTOTYPE*, and 0.45 for *IP*. For a relatively high *LT* the clustering coefficient approaches approximately to 0.7. A possible explanation can be that contribution of at least three people happens often in scientific collaboration teams (Newman et al., 2001). Therefore, every collaboration that has three or more co–authors increases the clustering coefficient significantly.

Centrality measures indicate the importance of nodes in the network. We measure betweenness centrality and closeness centrality to analyze datasets. For a node, the betweenness is defined as the total number of shortest paths between every pair of individuals in the network which pass through the node (Brandes, 2001). In other terms, it highlights collaborators who act as a bridge between different groups in a network.

Moreover, closeness centrality defines the closeness of a node to other nodes by measuring the average shortest path from that node to all other nodes within the network. Hence, the more central a node is, the closer it is to all other nodes (Sabidussi, 1966). All three datasets reach the highest closeness centrality after a certain threshold. However, each dataset reflects a considerably different growth function, such that *IP* follows a linear function after each evolution, *PROTOTYPE*, and *PAPER* are growing exponentially.

### 4.3. Community Detection Analysis

We imply *Louvain* community detection algorithm to evaluate *LT* on *collaboration score*. We extract communities of each network and measure the modularity and number of clusters. The modularity of communities illustrates the strength of connected nodes inside the same community compare to the community of a random graph (with the same size and average degree). The higher the modularity, the more the network is closer to a well-shaped community structure.

**Figure 3:**
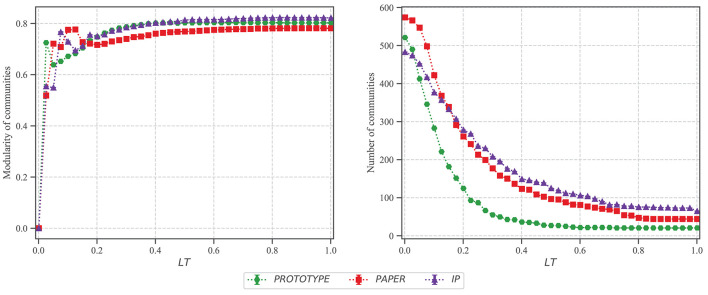
Community detection analysis after implying *Louvain* algorithm on networks produced with different *LT* values. The community modularity score, and the number of clusters are the average of 200 experiments for 41 data points. The error bars are not visible because the standard error is very small.

Figure 3 shows the average results of 200 experiments on each dataset including error bars. The figure shows that the modularity of all three datasets converges to relatively a high score of approximately 0.7 after a certain *LT*. It indicates that the produced collaboration networks have well–defined community structure compare to the random network of the same size. As illustrated in this figure, increasing *LT* does not affect the modularity after a particular point. For the lower *LT* (<0.4), as also shown in Figure 2 networks have a considerably lower density, thus, they are sparse. However, the score increases exponentially and becomes steady for all three datasets for *LT*>0.4. On the other hand, increasing *LT* decreases the number of communities considerably. When networks are sparse (i.e., *LT* ≤ 0.2) the number of communities is almost equal to the number of nodes.

Moreover, as illustrated in Figure 3, the modularity score increases significantly even for the low values of *LT* and reaches to its highest value before it decreases and becomes steady. On the other hand, the number of communities exponentially decreases. Therefore, the network obtained from *LT* <0.2 has an extremely high number of communities. In a particular case for *PROTOTYPE*, the modularity increases and becomes steady with *LT*>0.4, and similarly the number of communities become constant (= 22) with *LT*>0.5. Furthermore, considering the growth of metrics for *PROTOTYPE* from Figure 2, all metrics are constant with *LT*>0.4.

## 5. Discussion and Conclusion

The approach outlined in this paper infers collaboration networks of researchers within projects of an organization. Our method uses the features describing the collaborations of a research institute and quantifies them by applying a proposed *collaboration score* function.

Our results show that the quality of the detection of communities from the extracted collaboration networks can differ significantly by the choice of the linkage threshold. It turns out that a greedy increase of links and connections can lead to a noisy network structure where the *identity* of nodes could be affected by a large amount of superfluous connections. Consequently, our future work has to focus on the understanding of a networks preference toward a rich network while avoiding a noisy structure (Newman, 2018). Moreover, our experiments on the execution time of community detection indicate that increasing *LT* impacts the execution time of the algorithm. Hence, one option is to generate the network choosing a considerably low threshold while the modularity of communities is still at the highest possible value.

In this study we use a set of network metrics and the modularity score to evaluate communities of obtained networks. However, as future work we are looking at advancing our collaboration score model for network construction from relational data. Moreover, we consider identifying the optimum *LT* in order to recognize high quality communities within the obtained networks.

## Data Availability

The datasets generated for this study are available on request to the corresponding author.

## Author Contributions

SD developed the method and performed the computations and measurements. MB and PB were involved in planning and supervised the work. AP provided the datasets. MB, GD, and AP provided critical feedback.

### Conflict of Interest Statement

The authors declare that the research was conducted in the absence of any commercial or financial relationships that could be construed as a potential conflict of interest.

## Footnotes

^1^National Electronics and Computer Technology Center (NECTEC) (https://www.nectec.or.th/en/).
